# Book Review: Health Economics and Policy Challenges in Global Emerging Markets

**DOI:** 10.3389/fpubh.2017.00244

**Published:** 2017-09-12

**Authors:** Milena Jurisevic

**Affiliations:** ^1^Faculty of Medical Sciences, Department of Pharmacy, University of Kragujevac, Kragujevac, Serbia

**Keywords:** emerging markets, health economics, global health, medicine, health policy, BRICS, next eleven

The book entitled “Health Economics and Policy Challenges in Global Emerging Markets,” edited by Prof. Mihajlo (Michael) Jakovljevic MD, PhD (1) describes some of the key global health challenges at the beginning of the twenty-first century. Notable health economists in the field based in USA, Japan, China, and European countries (Germany, the Netherlands, Switzerland, Ireland, Serbia, Bulgaria, Poland, and Albania) affiliated to the some of the top ranked universities contributed with their diverse expert profiles in health economics, clinical medicine, public health, and population aging. The focus of the book is on the leading emerging countries and mature free-market economies that face with the similar challenges including BRICS nations (Brazil, Russia, India, China, and South Africa) and N-11 nations (Bangladesh, Egypt, Indonesia, Iran, South Korea, Mexico, Nigeria, Pakistan, the Philippines, Turkey, and Vietnam), North American region, Far East Asia, Western, and Eastern Europe. Regional health-care issues in these countries related to the population aging (2–4), health expenditures (5–7), health technology assessment (8, 9), insurance coverage (10–12), costs of pharmaceutical development (13, 14), economics of prosperity diseases (15–18), public health legislation (19), and caregiver assessment (20) are processed in seven chapters of the book.

The first chapter describes the trends in population aging and health expenditures in emerging markets of BRICS and N-11 nations thus complementing and expanding previous research in this field (21–24). The second chapter tries to answer the question “What can emerging markets learn from a public long-term care insurance system from a mature country?” by trying to find who benefits the most among different age cohorts by the change in policy using the pooled data of the National Survey on Life Insurance in Japan. Use of multi-criteria decision analysis (MCDA) in health technology assessment is described in the third chapter, with a special emphasis on international experience and practical lessons from the real-world MCDA models. Bulgarian experience in incorporation of MCDA into health technology assessment, along with the description of the weaknesses of this process, substantially adds to the current literature on this issue (25–27). Willingness-to-pay for a new pharmaceutical is a focus of the fourth chapter of the book through an example of the study, which sought to provide evidence for making a decision on whether a new pharmaceutical for insulin therapy should be included in the benefit list of social health insurance in Germany. The fifth chapter discusses the role of the health economics in psychiatry and non-epileptic attack disorder (NEAD) with its uniqueness characterized by crossing the boundaries of emergency medicine, neurology, and psychiatry. Study described in this chapter more specifically dealt with calculation of an estimate of the prevalence and incidence of NEAD along with its economic burden in Ireland. Attempt to define the key challenges and needs in terms of legislation of the public health system in Poland is presented in Chapter 6 based on the analysis of the collected opinions and positions of Polish public health system stakeholders. The final chapter in the book, Chapter 7, aimed to provide an example of how to quantify family caregiver burden in traditional East-Asian societies.

As can be seen from this brief outline of the book, combined efforts of the broad spectrum of the health economists have greatly contributed to the quality and comprehensiveness of the book. Various aspects of the current health economics and health policy challenges in emerging markets were covered and strategies to cope with these issues were analyzed. Intended themes are presented in an organized and progressive manner, and each chapter contains an abstract and a thorough list of references. Overall, Editor and contributors have made a remarkable effort in assembling this book, and the readers will certainly benefit from the different perspectives presented in the book. I would like to highly recommend it to the broader target auditorium of policy makers, health-care professionals, and lay audience regardless whether their primary career background is dominantly academic, medical care, or industry based.

## Author Contributions

MJ is responsible for the design, writing the article, critical revision, and final approval of the manuscript.

## Conflict of Interest Statement

The author declares that the research was conducted in the absence of any commercial or financial relationships that could be construed as a potential conflict of interest. The reviewer RDR and handling Editor declared their shared affiliation.
